# Tunable Graphene-Based Plasmon-Induced Transparency Based on Edge Mode in the Mid-Infrared Region

**DOI:** 10.3390/nano9030448

**Published:** 2019-03-17

**Authors:** Heng Xu, Zhaojian Zhang, Shangwu Wang, Yun Liu, Jingjing Zhang, Dingbo Chen, Jianming Ouyang, Junbo Yang

**Affiliations:** 1 College of Liberal Arts and Sciences, National University of Defense Technology, Changsha 410072, China; xuheng1995810@163.com (H.X.); 376824388@sjtu.edu.cn (Z.Z.); shangwuahab@sina.com (S.W.); study25@163.com (J.Z.); iqiy2019@163.com (D.C.); 2 College of Physics and Electronics, Hunan University, Changsha 410006, China; liuyun@hnu.edu.cn

**Keywords:** graphene, plasmon-induced transparency, edge mode, electric field distribution

## Abstract

A monolayer-graphene-based concentric-double-rings (CDR) structure is reported to achieve broadband plasmon-induced transparency (PIT) on the strength of edge mode in the mid-infrared regime. The theoretical analysis and simulation results reveal that the structure designed here has two plasmonic resonance peaks at 39.1 and 55.4 THz, and a transparency window with high transmission amplitude at the frequency of 44.1 THz. Based on the edge mode coupling between neighbor graphene ribbons, PIT phenomenon is produced through the interference between different (bright and dark) modes. The frequency and bandwidth of the transparency window and slow light time could be effectively adjusted and controlled via changing geometrical parameters of graphene or applying different gate voltages. Additionally, this structure is insensitive to the polarization and incident angle. This work has potential application on the optical switches and slow light modulators.

## 1. Introduction

Graphene, a single layer of carbon atoms gathered in a two-dimensional honeycomb lattice [1,2,3], has attracted significant attention in past few years owing to its excellent physical properties, such as ultra-high electronic mobility [4], extremely low loss [5], stable optical response [6] and more importantly, tunable surface conductivity [7,8]. According to the Kubo formula [9], the graphene surface conductivity could be effectively tuned by changing Fermi energy via electrical gating or chemical doping. Meanwhile, the short response time could make the ultrafast switching flexibly on the order of picosecond come true [10]. Therefore, tunable optical devices based on graphene have been widely developed in the nanoelectronics and optoelectronics domain [11,12,13,14,15], including optical modulators, photovoltaic cells and photodetectors.

Plasmon-induced transparency (PIT) based on graphene, as an electromagnetically induced transparency (EIT) analogue in the plasmonic system [16,17], has been a popular research hotspot due to widespread applications. As we all know, EIT is a quantum concept, coming from quantum coherence effect between atomic light excitation channels, which results in declining absorption of light at the atomic resonance frequency or even turns into completely transparent [18]. This phenomenon has promise for applications in label-free biological sensing [19], enhanced nonlinear effects [20] and slow light modulators [21]. However, its implementation is restricted by harsh experimental conditions such as the need for stable pumping, low temperature and high power laser systems [22,23,24,25]. Besides, the frequency of the transparent window is mainly limited in the spectrum because of limited energy interval [26]. Therefore, plenty of works about PIT have been investigated in the aspect of nanostructures due to operability at room temperature and wide operational bandwidth. Ever since S. Zhang et al. [27] demonstrated a π-shaped metamaterial (MM) structure to research PIT, varieties of MMS structures have been emerging endlessly, including split-ring [28], cut wires [29], U-shape [30] and various combination structures [31,32,33]. Recently, Z. Zhang et al. [34] studied a hybrid metal-graphene MM to achieve the active control of broadband PIT in the terahertz (THz) region. L. Han et al. [35] demonstrated anisotropic PIT in black phosphorus nanostrip trimer realizing wide range adjustment at the mid-infrared (MIR) region. L. Jiang et al. [36] reported the low-threshold optical bistability of reflected light by using the multilayer structure at THz frequencies. The realization of the PIT effect is usually achieved by destructive interference between the bright and dark modes via near-field coupling [37]. However, most of structures are too complicated to be fabricated easily and they commonly concentrate on switching the amplitude of a narrowband PIT. Moreover, plenty of structures only support surface plasmonic mode rather than edge plasmonic mode.

In this paper, we propose a concentric-double-rings (CDR) monolayer-graphene structure to achieve the effective control of broadband PIT based on edge mode. Via near-field coupling, the broadband PIT could be realized by destructive interference of different modes. This structure could not only manipulate the bandwidth of the transparent window by changing the radius of rings in the manufacturing process but also control the frequency position of the PIT window in the spectrum by tuning the Fermi energy of graphene. Additionally, it possesses the polarization-insensitive and large angle tolerance properties. Meanwhile, both the region and capability of slow light also could be tunable via Fermi energy of graphene. This work offers possible applications at tunable MIR functional devices, such as optical switches and slow light modulators.

## 2. Structures and Methods 

**Figure 1:**
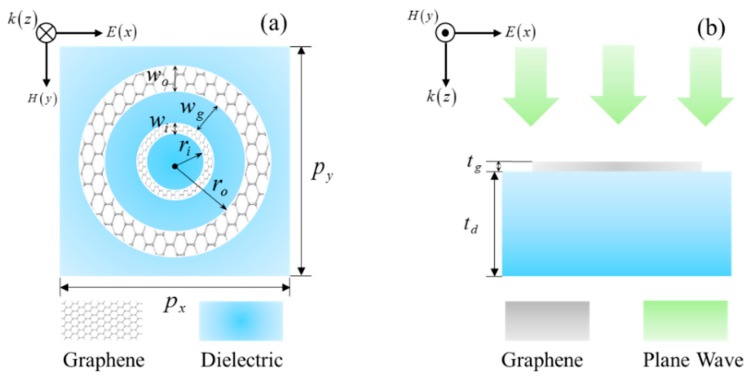
Schematic of the geometry of the proposed monolayer graphene-based concentric-double-rings (CDR) structure. (**a**) The top view of the unit cell: the periodic graphene nanostructure arrays with radius of inner-ring *r_i_* = 90 nm, width *w_i_* = 10 nm, radius of outer-ring *r_o_* = 148 nm, width *w_o_* = 30 nm, and period *p_x_* = 400 nm and *p_y_* = 400 nm. (**b**) The side view of the unit cell: the thickness of graphene *t_g_* = 1 nm, the thickness of dielectric *t_d_* = 2 μm.

Figure 1a,b denotes the geometry of the proposed CDR structure, which consists of the patterned graphene arrays and dielectric substrate, and the corresponding detailed geometric parameters are given in the caption. This structure is periodic in the *x* and *y* directions with periodicity 400 nm, and the periodic graphene pattern is composed of two parts: outer ring (OR) and inner ring (IR), laid on the dielectric substrate with the refractive index of 1.6. The width of gap *w_g_* between OR and IR is 48 nm, which could be adjusted in the fabrication process according to the requirements. The OR and IR of graphene have the same material properties except the size. Additionally, we assume a plane wave at the MIR region vertically impinges on the CDR structure along the negative *z* direction and the incident electric field polarized along the *x* direction.

The complex surface conductivity of monolayer graphene could be derived by the Kubo formula [9], which consists of intraband and interband transitions and describes as following:

(1) $$\sigma_{s}\left(\omega ,\mu_{c},\Gamma ,T\right)=\sigma_{intra}+\sigma_{inter}.$$

**Figure 2:**
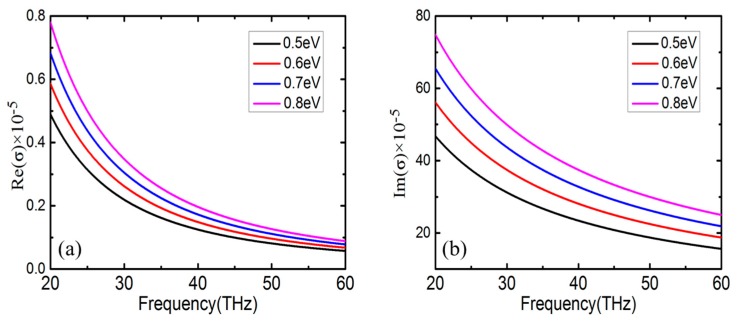
Diagram of graphene conductivity (**a**) real part (**b**) imaginary part at different Fermi levels.

In the random-phase approximation, the specific expressions are

(2) $$\sigma_{intra}=-j\frac{e^{2}k_{B}T}{\pi \hbar^{2}\left(\omega -j2\Gamma \right)}\left[\frac{\mu_{c}}{k_{B}T}+2\ln\left(\exp\left(-\frac{\mu_{c}}{k_{B}T}\right)+1\right)\right],$$

(3) $$\sigma_{inter}=-j\frac{e^{2}}{4\pi \hbar }\ln\left[\frac{2\left|\mu_{c}\right|-\left(\omega -j2\Gamma \right)\hbar }{2\left|\mu_{c}\right|+\left(\omega -j2\Gamma \right)\hbar }\right],$$

herein, *e* is the charge of an electron, *k_B_* is the Boltzmann’s constant, *T* is the absolute temperature in Kelvin and the *ℏ* = *h*/2π is the reduced Planck’s constant. Γ and *μ_c_* is are the phenomenological scattering rate and Fermi energy level, respectively. *τ* = 1/2Γ is the relaxation of electrons, which is governed by *τ* = *μ_m_μ_c_/ev_f_*^2^. In this equation, *μ_m_* is the carrier mobility and *v_f_* denotes the Fermi velocity. *ω* is the radian frequency of the incident wave. Moreover, if the photon energy is far less than the Fermi energy, the interband contribution consequently could be neglected according to the Pauli Exclusion Principle, and only the intraband part will be considered [38]. The real and imaginary parts of the conductivity of graphene are calculated in Figure 2 with regard to different Fermi energy levels. Obviously, both the real and imaginary parts of the conductivity of graphene decrease with the increase of the incident wave frequency. The effective permittivity of graphene is

(4) $$\varepsilon_{g}=1-j\frac{\sigma_{s}\left(\omega ,\mu_{c},\Gamma ,T\right)}{\varepsilon_{0}\omega \Delta },$$

where *ε*_0_ is permittivity of vacuum, Δ is the thickness of graphene. In this paper, the carrier mobility is fixed as 1.0 × 10^4^ cm^2^/V/s, the environment temperature is 300 K, and the Fermi velocity is set as 1.0 × 10^6^ m/s.

In this study, the numerical simulations were carried out by the finite-difference time domain (FDTD) method and we used the software called FDTD Solutions to simulate the features of proposed CDR structure. The FDTD method is a well-known method to solve Maxwell’s equations in the time domain. The Maxwell’s equations in a source free region are given as [39]:

(5) $$\nabla \times E=-\mu \frac{\partial H}{\partial t},$$

(6) $$\nabla \times H=\varepsilon \frac{\partial E}{\partial t},$$

where **E** is the electric field and **H** is the magnetic field. *μ* and *ε* are the permeability and permittivity of the medium respectively. According to the material parameters and initial conditions, the electro-magnetic field’s quantities in each space-time point could be evaluated by solving Maxwell’s equations. In the simulations, the periodical boundary conditions (PBC) is employed for a unit cell in the *x* and *y* directions and the perfectly matched layer (PML) is applied in at the top and bottom of the structure along the *z* direction. In order to get more accurate calculation results, we divided mesh grids towards unit cell with d*x* = 2 nm, d*y* = 2 nm, and d*z* = 1 nm. Then the Frequency-Domain Field and Power (FDFP) monitor was utilized detect the transmitted power at the bottom of structure. 

## 3. Results and Discussion

**Figure 3:**
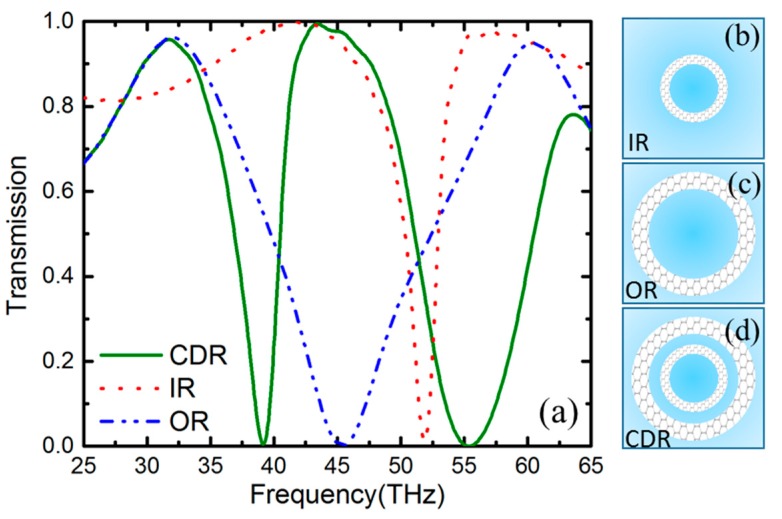
(**a**) Transmission spectrum corresponding to the unit cell with an inner ring (IR), an outer ring (OR) and a concentric-double-ring (CDR). The Fermi energy of graphene is fixed at 0.64 eV and the refractive index of dielectric is set as 1.6 in the simulations. (**b**–**d**) Structural representation of IR, OR and CDR, respectively.

In order to demonstrate the PIT effect, we numerically calculated the transmission spectrum of the unit cell with different patterned graphene structures. As shown in Figure 3a, the red dot line, blue dash line, and olive solid line respectively represent the transmission spectrum of the unit cell corresponding to only IR (Figure 3b), only OR (Figure 3c) and CDR (Figure 3d) structures, and the other geometric parameters keep the same as in Figure 1. It is obvious that the plasmonic resonances of the IR and OR structure are excited at different frequencies, explained by the Fabry–Perot model. When the frequency is 51.9 THz, the transmission of the IR structure is close to 0, but for the OR structure, the lowest transmission appears at 45.4 THz. For the proposed CDR structure, there are two plasomonic resonance peaks at 39.1 and 55.4 THz observed, and a transparency window is found at the frequency of 44.1 THz. Particularly, the amplitude of transparency window is very high. There exists light transmission for CDR structure at the frequency band where there is no light transmission for OR structures. Therefore, the PIT phenomena could be produced in the unit cell of CDR structure by combining IR and OR. The reason why this PIT phenomenon appears is the interference between the bright and dark modes via near-field coupling, which could be explained by the plasmonic hybridization model (PHM) in theory [40].

**Figure 4:**
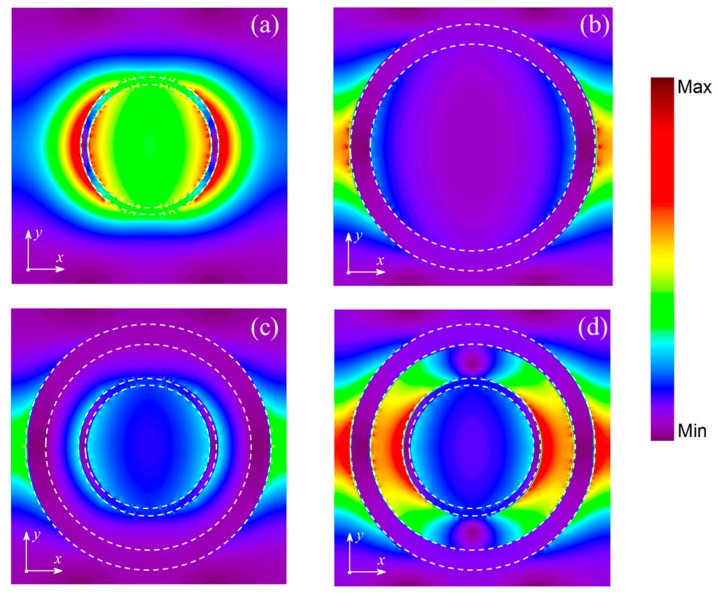
The electric field distributions at (**a**) 51.9 THz when the structure of graphene is individual IR, (**b**) 45.4 THz when the structure of graphene is individual OR. The electric field distributions at (**c**) 55.4 THz, (**d**) 39.1 THz when the structure of graphene is CDR.

**Figure 5:**
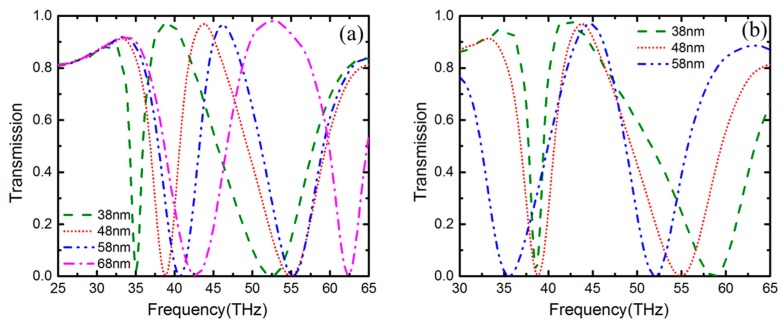
Transmission spectrum corresponding to different widths of the gap, 38, 48, 58 and 68 nm, respectively. (**a**) Keeping the geometric parameters of OR same and changing the radius of IR. (**b**) Keeping the geometric parameters of IR same and changing the radius of OR.

To further clarify the mechanism of the transparent window, three sets of unit cells with different structures were investigated. The corresponding electric field distributions at plasomonic resonance peaks are shown in Figure 4, where the absorption is extremely strong. According to Figure 4a,b, we could find that the electric field is mainly distributed at the edge of the ring-shaped graphene. Besides, there exists electric field interaction in the gap between IR and OR, which we could obtain from Figure 4c,d. Therefore, the PIT phenomenon is produced by interference between the bright modes and the dark modes from IR and OR via near-field coupling based on the corresponding edge mode, which can be explained by PHM. The interference is closely related to the structure parameters of the unit cell and directly have an influence on the position of frequency and the full wave at half maximum (FWHM) of the transparency window. The width of gap *w_g_* between IR and OR is the main factor we would consider firstly. As shown in Figure 5, in this case the width of IR *w_i_* and OR *w_o_* remain unchanged, Figure 5a is the transmission spectrum when changing the radius of IR but keeping the radius of OR the same as Figure 1, and Figure 5b is the transmission spectrum when changing the radius of OR but keeping the radius of IR the same as Figure 1. When we change the outer radius of IR or the inner radius of OR to increase the width of the gap *w_g_*, there is a blueshift in the transmission. The FWHM of transparency window also changes with the width of the gap *w_g_* increasing. For instance, the FWHM is broadened from 7.9 to 13.2 THz when the width of the gap increases from 48 to 68 nm in Figure 5a.

**Figure 6:**
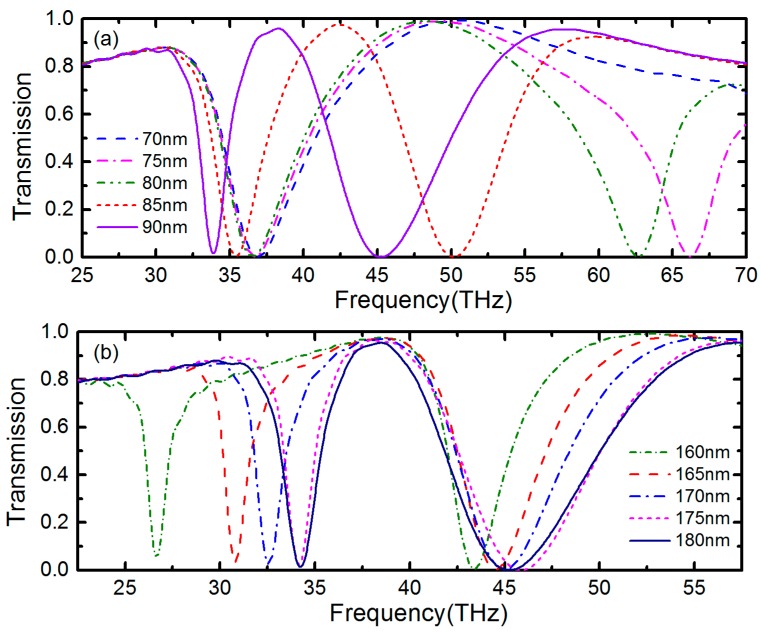
Transmission spectrum for (**a**) different inner radii of IR and (**b**) different outer radii of OR with other geometrical parameters unchanged.

Then, the relationships between transmission spectrum and width of IR and OR are also demonstrated. As depicted in Figure 6, we maintain the other geometrical parameters unchanged, including the width of the gap, and only change the inner radius (*r_i_*) of IR or outer radius (*r_o_* + *w_o_*) of OR. As the inner radius of IR decreases, the frequency of transmission window experiences a blue shift and the FWHM of it broadens, over 20 THz at the inner radius of 80 nm. The amplitude of transparency window also increases with the inner radius of IR decreasing. When the inner radius of IR is less than 75 nm, the PIT phenomenon disappears from transmission spectrum. When we change the outer radius of OR, the transparency window also could be adjusted, including frequency, FWHM and amplitude. Additionally, we could find that the width of OR mainly influences high frequency plasomonic resonance peak and the width of IR mainly influences low frequency plasomonic resonance peak. 

**Figure 7:**
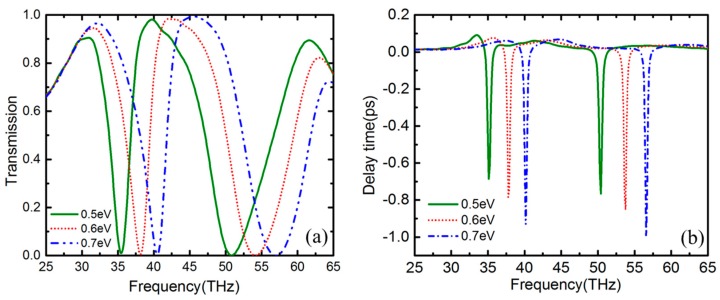
(**a**) Transparency spectrum for different Fermi energy of graphene from 0.5 to 0.7 eV. (**b**) Slow light region corresponding to different Fermi energy of graphene.

The frequency tunability of PIT is a significant important feature in practical application. The imaginary part of the conductivity of graphene determines the spectral shift of the resonance, and the real part controls the amplitude modulation of the resonance [41]. Hence, the transparency window could be adjusted indirectly by tuning the Fermi level via the applied gate voltage or chemical doping. Figure 7a plots the transmission spectrum for different values of *μ_c_*, 0.5, 0.6 and 0.7 eV, respectively. As the Fermi energy increases, the frequencies of the transparency window tend to exhibit a blueshift. However, the FWHM and amplitude of transparency window for different Fermi energy are approximately same. As shown in Figure 7b, slow light, as one of the most important applications of PIT, is also studied for different Fermi energy. The slow light could be qualified by the delay time *τ_g_* [42]:

(7) $$\tau_{g}=\frac{d\psi \left(\omega_{il}\right)}{d\omega_{il}},$$

where *ω_il_* is the circular frequency of incident light, and *ψ*(*ω_il_*) is the transmission phase shift from the light source to calculated point. When the Fermi energy is at 0.7 eV, there is a slow light region due to the extreme dispersion within the transparency window. Additionally, the slow light region experiences a redshift with the Fermi energy decreasing. As a result, the frequency of transparency window and slow light could be electrically controlled.

**Figure 8:**
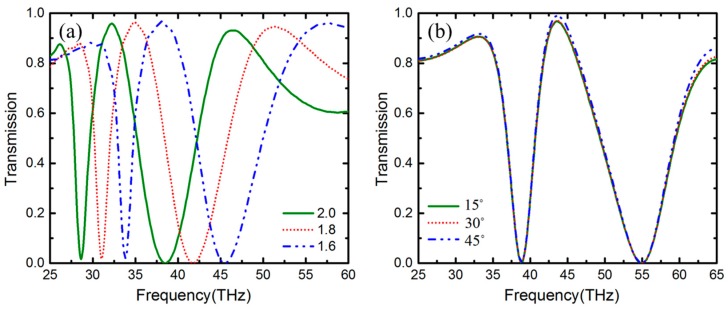
Transmission spectrum corresponding to (**a**) different refractive indexes (**b**) different incident angles.

Furthermore, we investigated the effect of refractive index of substrate on the transmission spectrum. In Figure 8a, we maintained the structure parameters unchanged and merely varied the refractive index of substrate from 1.6 to 2.0 successively. It is clear that the transmission spectrum exhibits a redshift with the increasing index. Additionally, the FWHM of the transparency window becomes narrower. In addition, the PIT phenomenon under oblique incidence is researched in Figure 8b. The incident angle is defined as the angle between the incident wave and negative *z*-direction, varying from 15° to 45°. It is obvious that the transmission spectrum is independent of the incident angle. Besides, due to the central symmetry of the structure, the transmission is insensitive to the polarization angle.

## 4. Conclusions

To conclude, we have proposed a monolayer-graphene-based CDR structure to realize broadband PIT on the strength of edge mode in the mid-infrared spectrum. The frequency and bandwidth of the transparency window could be effectively adjusted and controlled via changing the geometrical parameters of graphene or applying different gate voltages. The simulation results show that the structure has two plasomonic resonance peaks at 39.1 and 55.4 THz, and a transparency window with high amplitude at the frequency of 44.1 THz. The transmission spectra experience blue shift when the Fermi energy of graphene increases or refractive index of the substrate decreases. The bandwidth of the transparency window is broadened by increasing the width of the gap, IR or OR. Besides, the transmission is insensitive to polarization angle and incident angle. We believe that our research could be used in many future device applications, such as optical switches and slow light modulators in the mid-infrared region.
